# Identification and engagement of naturally occurring retirement communities to support healthy aging in Canada: A set of methods for replication

**DOI:** 10.1186/s12877-022-03045-z

**Published:** 2022-04-22

**Authors:** Vincent G. DePaul, Simone Parniak, Paul Nguyen, Carri Hand, Lori Letts, Colleen McGrath, Julie Richardson, Debbie Rudman, Imaan Bayoumi, Helen Cooper, Joan Tranmer, Catherine Donnelly

**Affiliations:** 1 School of Rehabilitation Therapy, Queen’s University, Kingston, Canada; 2 Health Services and Policy Research Institute, Queen’s University, Kingston, Canada; 3 Institute of Clinical Evaluative Sciences, Queen’s University, Kingston, Canada; 4 School of Occupational Therapy, Western University, London, Canada; 5 School of Rehabilitation Sciences, McMaster University, Hamilton, Canada; 6 Department of Family Medicine, Queen’s University, Kingston, Canada; 7 Oasis Senior Supportive Living Inc, Kingston, Canada; 8 School of Nursing, Queen’s University, Kingston, Canada

**Keywords:** Healthy aging, Health planning, Housing, Living environments, Community development

## Abstract

**Background:**

Naturally occurring retirement communities (NORCs), unplanned communities with a high proportion of older adult residents, offer a model to support older adults to age well in place. The aim of this paper is to provide a comprehensive description of the methods used to identify and engage NORCs appropriate for the development of supportive service programming in Canada.

**Methods:**

Three steps were used to identify and select NORCs in which to develop supportive service programming including: 1) identification of potential NORCs using Canadian Census Dissemination Areas, the Ontario Marginalization Index and Google Maps, 2) engagement of property owner/manager to determine the availability of common space for communal programming and willingness of the owner to support programming and, 3) engagement of older adult residents within the NORC to co-design programming.

**Results:**

Four cities in the south-east, south-central, and south-west of Ontario, Canada were identified to develop NORCs with supportive service programming. Using the methods described, six NORCs were identified, landlords and older adult residents were engaged, and programs initiated between April 2018 and March 2019. The sites included two private high-rise apartments, a city-owned low-rise subsidized apartment complex, two multi-building private high-rise complexes and a mobile home community. An average of 35 (min 20, max 78) older adult members were engaged in an average of 20.5 unique activity sessions at each site per month. On average, social (54%) and physical activities (30%) were more common than nutritional (10%) and knowledge-sharing (8%).

**Conclusions:**

The increased prevalence of unplanned, geographically-bound NORCs creates an opportunity for governments, social and health service providers and policy makers to support healthy aging in their communities. Our experience with the creation of six new NORCs with supportive service programming provides a tested set of methods that can be applied in other communities.

In 2019, one out of six (17.5%) Canadians were 65 years of age or older [1]. The vast majority of older Canadians (> 92%) live in their own home and want to remain in the community for as long as possible [2]. While the goal to age in place is common, aging well in the community can be challenging. In a Canadian survey approximately 20% of older adults (65 years or older) reported feeling a lack of companionship, left out or isolated from others; with this number rising to 25% for those 85 years or older [3]. Actual and self-perceived social isolation are associated with increased risk of mortality and morbidity, including cardiac disease [4], stroke [4], cancer [5], cognitive decline [6, 7], depression and anxiety [8, 9], reduced physical activity[10], and inadequate nutritional intake [11–13]. Lonely and socially isolated older adults are at increased risk of being admitted and readmitted to hospital [14, 15], staying longer in hospital, requiring admission to long-term institution-based care [16] and premature death [17]. As the population of Canada continues to age [1], it is essential that effective, scalable strategies are developed to support older adults to not only age in place, but to remain socially connected, healthy and active in the communities of their choice.

Naturally occurring retirement communities (NORCs) are defined as housing developments or communities that are not specifically planned or designed for older adults, but which over time come to house a high proportion of older adult residents. [18]. These communities can be further classified as vertical (e.g. rental apartment buildings) or horizontal (e.g. neighbourhoods of single-family homes) NORCs [19]. NORCs are ideally positioned to offer a solution to the challenges of aging in community by integrating programs designed to provide health and social supports tailored to the needs of a geographically-bound cluster of older adult residents. NORCs with targeted supportive service programming (NORC-SSPs) are associated with increased engagement in social and recreation activities, reduced levels of depression, and increased overall quality of life [20–22]. The first NORC-SSP described in the literature was established in a New York City housing community (Penn South Houses) in 1986 and expanded to other vertical NORCs (apartment complexes) in New York, New Jersey, Ohio and other American states [23–25]. Although NORCs are increasingly prevalent [26], there has been limited exploration of NORCs and NORC-SSPs in the Canadian context. Significant between-country differences in the organization, funding and jurisdictional responsibility of health and social services [27], may limit the translation of US-centric literature to Canadian settings. In particular, a single-payor universal health insurance system, and a combination of federal and provincial pension and benefits programs, are proposed to provide a safety net, with particular protection for seniors with lower socioeconomic status [28]. There has only been one example of a Canadian NORC-SSP described in the literature; the Cherryhill NORC in London, Ontario [21]. In 1996, the Cherryhill NORC, a community of 13 private apartment complexes with a majority of older adult tenants, was identified as an ideal location to establish onsite health-related programming using a participatory action research and community development approach. The Cherryhill experience provides valuable lessons regarding the maintenance and evolution of NORC-SSPs in the context of one Canadian city.

Despite the demonstrated benefits of these NORC-SSPs, little has been written to guide communities through the process of establishing a successful NORC-SSP. A number of papers have described the specific social services and health services programming within their NORC [29–33]. Other authors have described their experiences in tenant engagement and sustaining programming within a community [20, 21, 23–25, 34–40]. Essential to the success of any NORC-SSP is the initial step of identification and selection of the NORC itself – Where are these clusters of older adults located within a city or community? To date, only one paper has provided a methodology for NORC identification [41]. Rivera-Hernandez et al. (2015) described a systematic spatial analytic approach using geographic information systems to identify the location and temporal changes of NORCs in Ohio over 10 years. Investigators created and analyzed maps based on census data from the years 2000 and 2010 using United States (US) census tracts – each tract representing approximately 4000 residents (from 1,200 to 8,000)[42]. Their work provides a method suitable for other American jurisdictions to understand the geographical distribution of NORCs, potentially informing NORC-SSP planning in these areas. Unfortunately, differences in population density, and census methodology in Canada compared to the US, limits the application of their approach in this country. In Canada, census tracts include up to 10,000 residents, may represent a large geographic area, and are only used in cities with a core population of at least 50,000 people [43], excluding many smaller communities. Dissemination areas (DA) are a tighter geographical designation representing between 400 and 700 residents, that cover the entirety of Canada [44]. Use of the smaller DA designation allows for more precise identification of NORCs in Canada, and should better enable the planning of services to support these communities of older adults.

Oasis Senior Supportive Living Inc. (Oasis) is a unique NORC-SSP first developed in 2011 in Kingston Ontario through a partnership between the older adult residents of a midsize private apartment building, the landlord, the local Council on Aging, and regional health partners [45]. Similar to other NORC-SSPs, the aim of Oasis is to support older adults to live as long as possible in the home and community of their choosing. Programming focusses on promoting social connections, physical activity, and nutritional wellness of older tenants living in this mixed-age building. Oasis members identify preferences for programs and work with an onsite coordinator to implement these programs, including engaging local social and health service agencies to deliver group activities in a common space provided in-kind by the property owner. A key element of the Oasis model is the provision of a nutritional meal, delivered in a communal manner in the building’s common space. In terms of governance, members voice their concerns, desires and preferences in a monthly members meeting, and provide direction to a volunteer Board of Directors at monthly Board meetings. Core to the success of the Oasis model is the engagement of the landlord or property owner and staff, who supports membership recruitment, provides initial and ongoing access to common meeting spaces, and ensures building access for Oasis coordinator and partner agencies to deliver programming.

As health professionals, educators, and clinician researchers working in Kingston, Ontario, we were increasingly aware and intrigued by this model founded and directed by older adults in our community. Similarly, the Oasis model has been specifically cited by local and provincial governments as a uniquely successful response to the potential challenges of an aging population and an exemplar suitable for replication. Given its origin in our community, its history of success in the Canadian context, its recognition by the Government of Ontario, and interest of the older adults in collaborating, our research group partnered with the Oasis members and Board of Directors to successfully expand the model to six new NORCs in four Ontario jurisdictions and conduct a longitudinal evaluation in these communities. This work was funded in part by the Government of Ontario.

The aim of this paper is to provide a comprehensive description of the methods used to identify NORCs appropriate for the development of Oasis programs, and to initiate engagement of the older adult residents of these NORCs in the development of the specific Oasis community. Our intent is to use our experience with Oasis as an exemplar that can be applied for the development of similar NORC-SSPs in other communities.

## Methods

Successful implementation of Oasis NORC-SSPs requires careful selection of an appropriate site. For the Oasis expansion project, there were three primary steps to selecting sites for the Oasis model; 1) identification of potential NORCs or sites (e.g. apartment buildings) within NORCs, 2) engagement of property owner/manager and, 3) engagement of older adult residents within the NORC. Each of these steps included sub-steps and consideration of multiple factors. Once a site was confirmed eligible through the above process, Oasis site development through resident co-design began (Step 4). This paper will focus on the first 3 steps, the identification and confirmation of sites.

### Step 1: Identification of NORCs

Informed by methods described by Rivera-Hernandez et al. (2015) [41], Canadian Census (2016) data were used to identify geographical areas with high percentage of older adults (≥ 25% aged 55 years or older) in four cities/towns in Ontario. Although Rivera-Hernandez and colleagues used census tracts (approximately 4000 residents) [41], we utilized Dissemination Areas (DAs), the smallest standard geographical area for which census data is organized, each including between 400 and 700 individuals. [44] These smaller clusters allowed for a more precise identification of NORCs to consider for Oasis implementation and rely on publicly available data. In addition to age, additional census-based characteristics were considered in the selection of potential sites. Socio-demographic factors are known to significantly impact health and wellbeing. For example, individuals with lower socio-economic status are higher risk to have worse health outcomes and reduced life expectancy compared to those with greater resources. [46] We therefore applied the Ontario Marginalization Index [47, 48] to DAs that met the criteria as a NORC. The Ontario Marginalization Index provides a comprehensive analysis of populations across the province, sectioned into the DAs that correspond to the Canadian Census [47]. A mean marginalization index for each DA is calculated by averaging the quintile scores for four dimensions; residential instability, material deprivation, dependency, and ethnic concentration.

In order to explore the influence of these socioeconomic factors on Oasis implementation, we purposely identified NORCs with low, mid and high marginalization indexes.

Once a DA was identified as appropriate based on age characteristics and marginalization index, we used open-access web-based mapping software (Google Maps) to locate apartment buildings or other high-density living environments within each eligible NORC in the four cities. Consistent with the definition of a NORC, retirement homes, long term care facilities, planned retirement villages, and seniors-exclusive apartments were excluded, as these are intentional older-adult oriented living environments. A list of potential sites was generated including name of building/property, address, website (if available), property owner (landlord) and contact information. Potential sites were also characterised in terms of proximity and walkable-access to amenities (e.g. grocery stores, library) using Google Maps, Google Street View, and Walk Scores [51]. Finally, for buildings that met initial eligibility criteria, we physically visited the building or community sites to identify and understand the environmental characteristics and context of the potential site. These included proximity and maintenance of bus stops or shelters, distance between buildings in apartment complexes, and seasonal maintenance and accessibility of walkways surrounding the community. Site visits also gave context to location of sites that could not be identified on Google Maps. For example, a building may be a two-minute walk from the local library, but on visit, was discovered to be a two-minute walk across four lanes of traffic without traffic lights or sidewalks.

In addition to census data, site idenfication was informed by data gathered from local community-based health and social service providers. Specifically, the Home and Community Care service of the Local Health Integration Networks, the regional health authority in Ontario at the time of the study, was asked to identify apartment buildings or other concentrated communities that had relatively high rates of home care service use (professional and non-professional). These data provided an indication of functional abilities in the NORC and the potential need for supportive service programming. High home care service use was considered among the other demographic data when prioritizing communities as potential sites for Oasis programming.

### Step 2: Engaging the Property Owner/Manager

Once a potential site was identified, property owners were contacted by email, phone or in-person visit. Meetings were organized with the representative at the building, typically a manager or superintendent, to introduce the Oasis concept and the potential to develop an Oasis community at their site. In addition to introducing Oasis, the intent of this step was to confirm: 1) the presence of a high proportion of older adults, 2) the presence and availability of common space to host Oasis programming, 3) the willingness of the property owner to support the Oasis program by allowing access to the site to engage with the older adult residents, and provide space as an in-kind contribution and 4) whether structured programming was already being offered in the building. If a site was already operating extensive social, exercise or other supportive programming, it was not pursued further as a potential Oasis site. These visits often included a tour of the premises, with specific focus on the available communal spaces, ensuring adequate space for at least 10 individuals to gather at one time. The presence of a kitchen, fitness facilities, or outdoor shared space was noted but not a requirement. Once these criteria were confirmed, property owners identified and connected us with a tenant, or group of older adult tenants considered to be informal leaders within the building or community. In one case (Oasis 5) where the property owner was not aware of such a person or group, we proceeded directly to Step 3 of the process – engaging residents.

At the stage of engaging with the property owner, owners with more than one building often identified additional potential buildings under their management that might be appropriate for an Oasis program. Reasons for suggesting these buildings were typically related to a known high proportion of older adult tenants, the presence of an individual or group of tenants who had tried to organize social activities in the past, and/or the presence of space for programming. In some cases, these buildings were already on our list of potential sites; however, in other cases, these were new sites that we added to the list to be considered.

### Step 3—Engaging the Residents

**Fig. 1:**
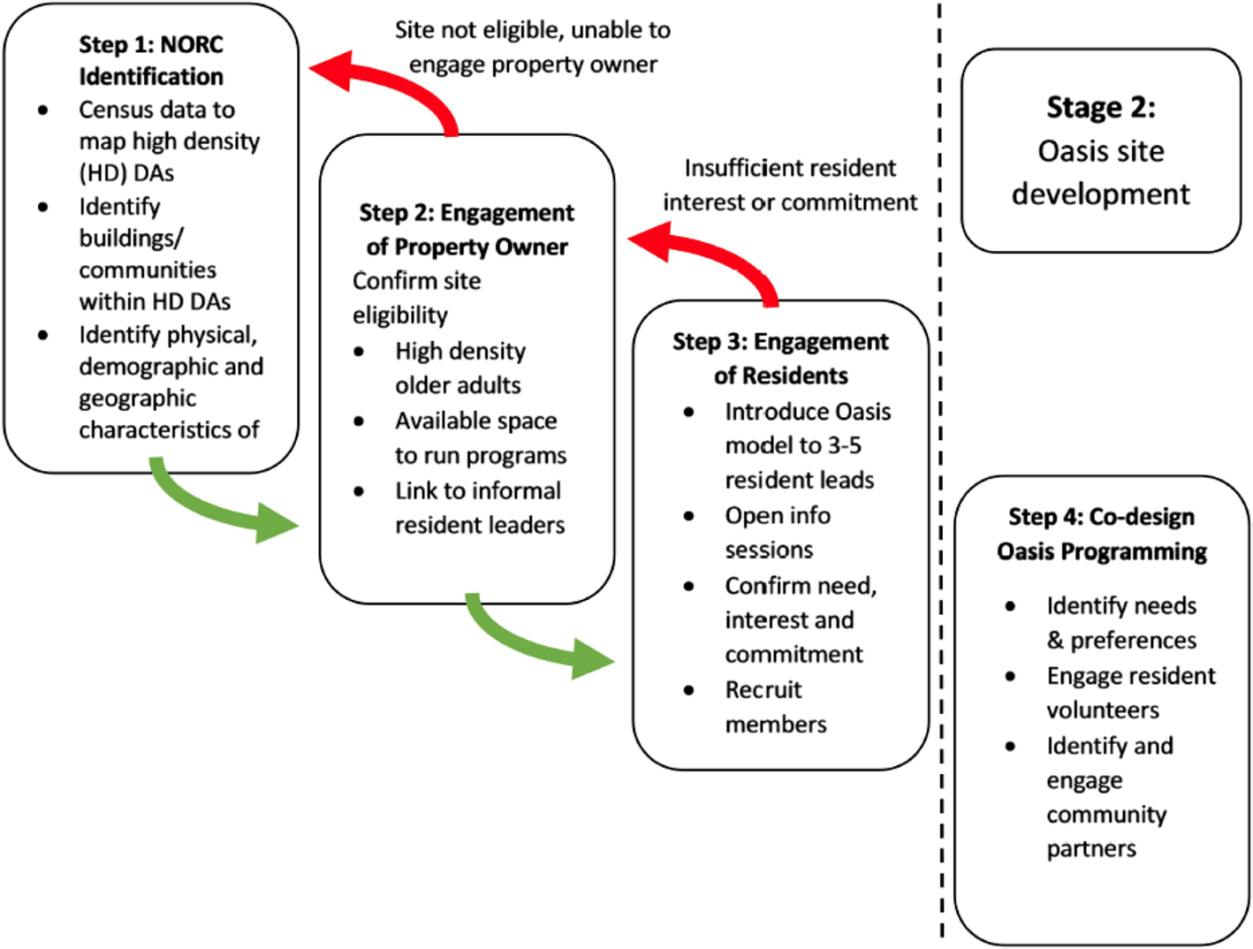
Steps for NORC identification and engagement

The next step to confirming an Oasis site was ensuring that residents in the building/community perceived a need and were committed to co-design and participate in Oasis programming at their site. As previously described, at most sites we initially met with the informal leaders identified by the property owner. In some but not all cases, these individuals were part of an existing residents’ social committee. A further method of resident engagement was to host informal information booths in the lobbies of selected NORCs, to meet residents, share brief information about Oasis, and begin to develop relationships with community members. An information session for the broader community of residents was then organized in the communal space with the assistance of the resident contacts and property owners/managers. This session included a brief formal introduction of the Oasis concept, the potential benefits, and roles and responsibilities of residents as members. This was followed by discussion of existing strengths of the community, and brainstorming regarding what Oasis may look like in their specific community. A list of interested residents with contact information was created and follow up meetings were organized for the purpose of Oasis program co-design and development. Recruitment of new Oasis members at each site was open-ended and ongoing, relying on word of mouth (e.g. bring a friend events), and posting of flyers in high traffic spaces such as the laundry room and community announcement boards. See Fig. 1 for graphic representation of the major steps for Oasis selection and resident engagement.

### Step 4: Co-design of Oasis Programming

Once a NORC site was deemed to be appropriate (Steps 1 and 2), landlord support (Step 2) and resident commitment was confirmed (Step 3), the Oasis Program co-design process was initiated. Consistent with Oasis’s core philosophy of promoting autonomy in older adults, decisions about content, format, and timing of programming were made by the older adult resident members of each Oasis site. Onsite Oasis coordinator facilitated member meetings on a biweekly to identify member needs, preferences, and prioirities. Similar to initial member engagement sessions, members generated ideas for programming in small group (using sticky notes) and in larger group discussions. Discussions were structured in relation to the three core Oasis programming pillars – social activity, physical activity, and nutrition-related activities. When possible, a consensus approach to final decisions was used. When consensus could not be reached, ideas receiving highest number of votes were moved forward to implementation. This process was iterative in nature. The Oasis Coordinator worked with a member-volunteer(s) to operationalize desired programming ideas. Programming was initiated and trialed, and reviewed at members committee meetings after a period of trial.

Process evaluation data collected included number of Oasis sites opened, member recruitment per site, and programming implementation. Program implementation metrics included average number of activities per month, number of activities in each of the three programming pillars (social, physical, nutrition), and member participation (average number of participants per activity session). Programming data was monitored daily by Oasis Onsite Coordinators, and submitted weekly to the Project Manager.

## Results

### NORC Identification Process

**Fig. 2:**
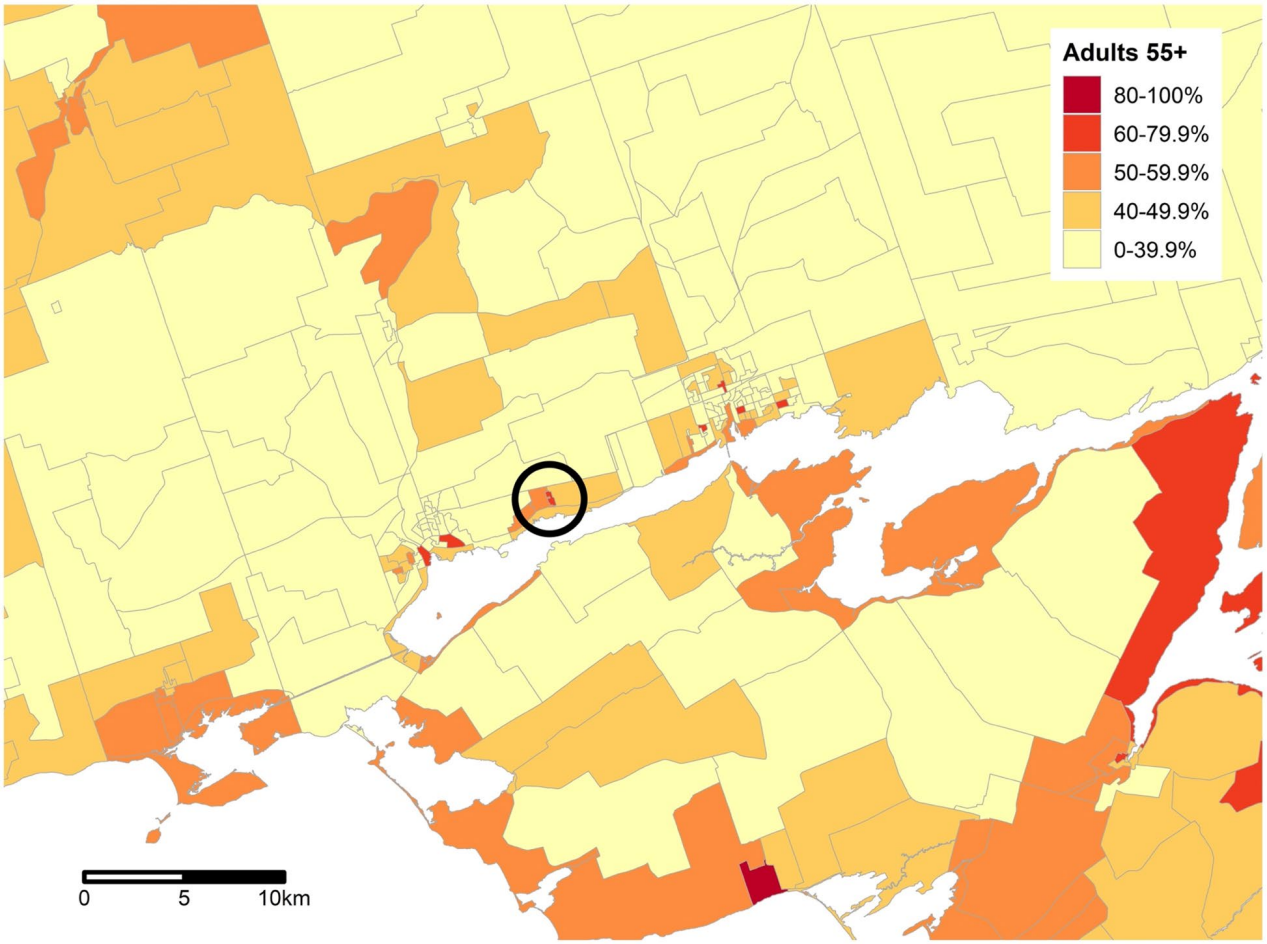
Map of Belleville and Quinte West displaying dissemination areas colour-coded by proportion of residents ≥ 55 years of age. *Note.* Geographical boundaries represent one dissemination area (DA). Colour coded to represent proportion of older adults, with darker areas with higher density. Darkest area within circle represents the NORC selected for Oasis program in one city

Four Ontario juridictions were selected for site identification and development: Kingston, Belleville/Quinte West, Hamilton, and London. These cities were selected to represent different sizes of communities, including larger cities (Hamilton (population 536,917), London (383,822) and midsize city (Kingston (123,798) and the smaller neighbouring jurisdictions of Belleville (50,716) and Quinte West (43,577). [49] These jurisdictions also differ in terms of population density, with London being the most densely populated with 913 people per square kilometre, and Quinte West being the most rural, and sparsely populated at 88.2 inhabitants per square kilometre. Finally, our research team had connections to a variety of stakeholders in these communities, including health and social service agencies, necessary for the implementation of the Oasis model. Using the described process, maps were created that identified DAs by proportion of older adults: one each for Kingston, Hamilton and London, and a combined map for Belleville and Quinte West. See Fig. 2 for map of DAs according to age distribution for Belleville and Quinte West map. As previously described, specific buildings and communities within the identified DAs were then identified using Google Maps.

Following the process outlined above, six NORCs were selected as Oasis program expansion sites. The six sites included two private high-rise (≥ 5 stories) apartment buildings in Kingston, one city owned, low-rise subsidized apartment complex in Kingston, two multi-building private high-rise apartment complexes (one each in Hamilton and London), and a mobile-home community in Quinte West. All sites were similar in that they met the criteria as a NORC – unplanned, mixed-age setting with higher proportion of older adults – and met our pre-set criteria for an Oasis Program – availability of shared space for programming, support of property owner/manager, and interest and commitment from older adult residents. Sites varied in terms of size (50 to 400 units), marginalization level (low to high), property type (tenant-owned, private rental units, subsidized rentals), NORC type—vertical (e.g. high-rise apartment building) and horizontal (e.g. mobile home community), and characteristics of surrounding area (high to low walkability to amenities). Characteristics of the six expansion Oasis communities are summarized in Table 1.

### Engaging property owners or managers

Following the mapping of DAs with a higher density of older adults, site identification required engaging and meeting with property owners and residents at potential sites. Within the larger property owner category, the engagement process varied by site. At one site, where we had an existing relationship with the property owner, a large landholdings company with multiple rental apartments, our initial contact was with a higher-level manager. This property owner was intimately aware of the Oasis model as they were a partner in the creation and ongoing support of the original Oasis community, and were interested in supporting Oasis in other buildings. At other private apartment building sites, our initial contact was with an onsite building superintendent, who then put us in touch with a building manager for further discussions. In all cases, this process took a number of interactions, by phone, email, and in-person. In the case of the city-owned building, our contact person was a Manager of Support Services, whose responsibilities extended beyond building maintenance or management to ensuring the residents were supported where possible. Although there was no existing programming running in this building, this person was very familiar with the residents, and their potential individual and collective needs related to continuing to live in the community. In the mobile home community, the ownership model was also unique. Each mobile home is owned by the residents, who rent the land from the park owner. Within the community there are two boards, a Residents’ Board, and a Recreation Board, each made up of residents from the community. As the planned Oasis programming was dependent on access to the recreation hall, our initial interactions were with the Recreation Hall manager and the Recreation Board. We then presented to and received support from the Residents Board.

### Engaging Older Adult Residents

Oasis is member-driven, and like other NORC-SSPs described in the literature, depends on engaging older adults at all phases of the development and ongoing operation of program. For all six sites, contact with residents followed our initial contact with the property owner or manager. At five of the six sites, the property owner/manager connected us to one or more tenants, all older adults who they thought would be interested and helpful with next steps. At Oasis Site 1, members of an existing social committee took a leadership role, meeting with us and planning a larger information session with other residents. At Sites 2, 3, and 6, while there was no currently-functioning social committee, the individuals had all taken an informal leadership role in organizing a community building event (e.g. organizing a community barbeque or garage sale), or had advocated on behalf of other tenants to the landlord about community needs or issues. At Site 5, a core group of interested residents were recruited via an information table set up in the building lobby over a number of days. At the mobile home community (Site 4), the fact that residents sat on both the Recreation Board and Residents Board, meant that residents were involved in all initial discussions. The apartment building site with the formal social committee (Site 1) was also unique in that they were already very aware of the original Oasis community, and unbeknownst to us, had previously contacted the original Oasis Board President about setting up an Oasis in their building. At the two other Kingston sites, while some residents had heard of the Oasis, they were not as familiar with the details of the program. Residents at the sites outside of Kingston were not aware of Oasis, but understood and were equally receptive to the concept.

### Iterative Nature Of Site Identification Process

**Fig. 3:**
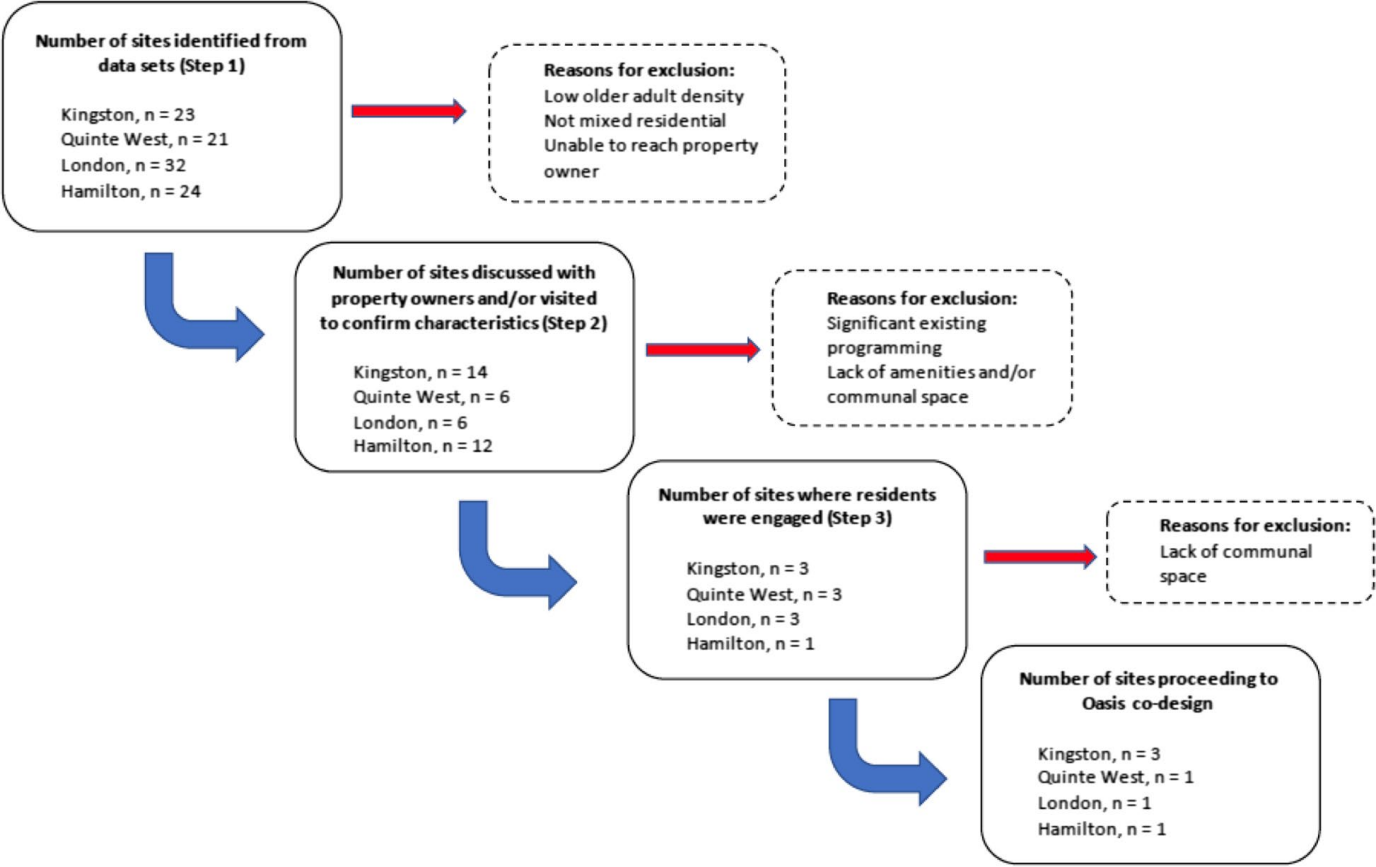
Number of sites assessed and considered at each stage of the identification process

As previously described, the process for selection of sites for Oasis NORC-SSPs included consideration of multiple variables. Although the process necessarily began with the identification of NORCs using the Census Canada age-described dissemination area maps, the process was iterative, tailored to specific communities and not necessarily linear. For each selected site, ten or more sites were considered and not selected based one or more of the previously described criteria (i.e. demographic make up, property owner/manager commitment, space availability, and finally interest and commitment of residents). In some cases, a potentially appropriate apartment building was identified within a DA with high proportion of older adults and evidence of communal meeting space, however the landlord was unresponsive to email, telephone and in person efforts to contact them, so the building was excluded. In another example, a three-building apartment complex was identified as a NORC based on census data, the landlord facilitated contact with older adult tenants, and tenants expressed a need for support services, and an interest in the Oasis concept. Unfortunately, the site lacked adequate common room space to accommodate Oasis programming. Efforts were made to identify alternatives to onsite space, including exploring use of a church hall across the road from the Apartment complex. An Oasis information meeting was held in this church space, and attended by a small group of motivated tenants. In the end, tenants expressed concerns about safety and convenience related to having to cross the street for activities, particularly in the winter months, ultimately requiring our team to search for a new site in this area. Although we were able identifying an alternate site, which has been a great success, it was difficult to have to move on from the original site as the need for supports remain unmet in this specific building complex. In Fig. 3, we provide a description of the number of NORC sites assessed and considered at each stage of the identification process.

### Site Participation and Programming

Site openings occurred in a consecutive manner – with Site 1 being the first to initiate programming in October 2018, and Site 7 the last, initiating programming April 2019. In-person activities were suspended at all sites on March 13, 2020 in compliance with provincial COVID-19 public health regulations. As of March 2020, regular Oasis programming was occuring in the six NORCs described in Table 1. Member recruitment occurred on a rolling basis. As of March 13, 2020, median membership was 35 older adults per site (min 20, max 78). Average age of all members was 74.8 years old (SD 5.3) with average age at each site ranging from 67 to 82 years old. Content and schedule of programming was determined through member meetings, facilitated by the Onsite Oasis Coordinator. An average of 20.5 unique activity sessions were held at each site per month (min average 18.8, max average 23.8) over the first 12 months of the program. An average of 7.7 members (min average 5.1, max average 13.8) participated in each session. Participation levels at all sites typically increased over the first 3 months, and stabilized over the remaining months. Types of activities initiated included social activities (median proportion per site = 54%; min 43%, max 79%), physical activity and exercise (median 30%; min 9%, max 41%), communal nutrition activities (median 10%; min 5%, max 18%), and knowledge-sharing events (e.g. guest speakers) (median 8%; min 7%, max 10%). Although occurring less frequently, participant numbers tended to be highest for meal and knowledge-sharing programs.

## Discussion

To our knowledge this is the first paper to provide an account of both the identification of NORCs and the establishment of a supportive service program within NORCs. Over the course of one year (April 2018 – March 2019), our group worked with residents and property owner/manager partners to successfully initiate six new Oasis NORC-supportive service programs in four Ontario cities. One year post-initiation, regular programming was running at all sites, with some between-site variations in number, type, and level of participation. The process for identification, engagement and initial development of each community described in this paper was tailored to fit the unique setting and context of each of the communities. This process was iterative and relied on multiple sources of data, including census data, open source mapping software, the knowledge of landlords and residents about the needs, group dynamics and informal politics of their communities. Detailing these steps is crucial to support the establishment of new NORC-SSPs by offering stakeholders a systematic, transparent and replicable process.

Much of what has been written in this field has focused on characterizing NORCs [51] and NORC-SSPs as a strategy to support aging in place [22, 24, 39, 40, 52]. By definition, NORCs evolve naturally [33], however the development of optimally connected communities, and the establishment of support services and structures within these naturally occurring communities requires planning and intention. As such, the first step in establishing an effective NORC-SSP is the careful selection of an appropriate NORC. Site selection requires consideration of factors at multiple levels; at a geographical level (macro), at the site and community level (meso), and a person level (micro). We are aware of a single publication that included description at the macro level, specifically a process for identification of prevalence and location of NORCs within the state of Ohio. [41] The intent of this paper was to describe the changes in geographic location and prevalence of NORCs in Ohio over time using census data. At a meso level, a few studies have emphasized the importance of the involvement of the property owner, either a public housing corporation as in the New York example [33], or a private landlord. At a micro level, a number of papers have described the process and challenges of ongoing engagement older adults within communities [38, 40, 53, 54] but typically fail to describe the initial steps of engaging residents. As far as we are aware, this is the first paper that provides a description of the multiple steps to site selection and engagement at the macro, meso and micro levels.

At its core, the process for creation of these Oasis NORC-SSPs is collaborative community development. Warburton et al. (2008) identified key factors for a successful collaboration, including the presence of an enabling context, diverse and skilled partners, clear operating and communication processes, shared vision and purpose, and sufficient resources [55]. While not explicitly driven by this framework, the described process of spreading the Oasis model to new communities considered and attended to all these factors, establishing a foundation for continued development of a successful collaboration. From a context perspective, sites were chosen based on apparent need (high density older adults, lack of other current programming), support of property owner/manager, and commitment of residents. Partners, including property owners and staff, residents, members of the original Oasis, the original Oasis Board, members of our research team, and to a lesser degree the regional health authority all brought unique and necessary knowledge, experience, skills, connections, and shared interest in supporting older adults to age well in their communities. We were explicit in the goals, roles and responsibilities to all stakeholders at all stages of the process. Efforts were taken to maintain clear and timely communication with and between our partners from holding open meetings with residents at new sites, provision of summary notes from each meeting to participants, to regularly sharing progress with property owners/managers, and the original Oasis members and Board.

A lack of previous attention in the literature to the initial identification and creation of NORC-SSPs may be related to the specific circumstances of the communities described. In the Penn South community in New York, the NORC-SSP was initiated with a partnership between a clinician (Social Worker) at a hospital in the neighbourhood and the Board of Directors of the public housing complex. Proximity, an existing relationship, and knowledge of the community make-up and needs led to the start of a comprehensive, state-funded multidimensional NORC-SSP. In the Cherryhill Healthy Ageing Program [21], the only other Canadian example described in the literature, the initial goal was to develop a health information education centre where residents were both learners and teachers within a shopping mall space in the community. Although the authors do not describe the initial site selection, they imply an awareness of the high number of seniors living in the area, making it suitable to set up this education-focused program. The aim of the Oasis Project was to spread an existing SSP model to six other NORCs in Ontario within a specific time period. We took a purposeful, criterion-based approach to identification and selection of the final sites for the project. Although our approach may not reflect the experience of all NORC-SSPs, we would argue that it is more applicable to the real challenges faced by municipal or provincial governments and agencies who need to find validated, scalable aging-in-place solutions that could be launched relatively quickly in their communities.

### Implications

This paper describes a process of identifying and engaging NORCs in order to establish needed programs and services and better support the well-being of older adults. The process described here has the potential for uptake by policy makers, program providers, researchers and community members interested in identifying and working with such communities. A combination of census and other forms of data as well as informal information from community partners is key to this process, to determine characteristics such as proportions of older adults, availability of resources such as space, and stakeholder support. Further research that involves applying this approach and reflecting on its utility, across a range of settings and purposes, could further refine the process described in this paper.

### Limitations

The aim of this paper was to describe a process for identifying and engaging NORCs in Canada in for the purpose of establishing NORC-SSPs to support older adults to age well in place. As such, it is beyond the scope of this paper to describe the effectiveness of this particular model of NORC-SSPs. Although our group has collected qualitative and quantitative data related to the impact of the Oasis program on a variety of health and health-related outcomes, these data will be summarized in subsequent publications. As previously noted, the vast majority of research related to NORCs has been completed in the United States. This paper is the first to describe a NORC identification and engagement process suitable for the Canadian context. Although the specific methods for identification relied on Canada-specific data and data categories, we believe that our experience with the other steps for identification and engagement would be broadly applicable in other jurisdictions.

## Conclusion

With the aging of Canada, the demographic make-up of communities have also changed. The increased prevalence of unplanned, geographically-bound NORCs creates an opportunity for governments, social and health service providers and policy makers to support healthy aging in their communities. We demonstrated that through the use of a systematic method for NORC identification and tenant and stakeholder engagement, it is possible to establish NORC-based programming and maintain engagement of older adult residents in six unique NORCs in the Canadian context. Our experience with the site selection and initiation of Oasis programs provides a tested methodology that can be applied in other communities.

**Table 1:** Characteristics of six sites selected for Oasis program development

	Site					
**Characteristic**	**Oasis 1**	**Oasis 2**	**Oasis 3**	**Oasis 4**	**Oasis 5**	**Oasis 6**
**Setting Description**						
City population	123,798	123,798	123,798	43,577	383,822	536,917
Population Density of city (per square kilometre)	274	274	274	88	913	481
Community type	High-rise apartment building	High-rise apartment building	Low-rise Apartment	Mobile Home Community	3-building High-rise Apartment complex	2 building High-rise apartment complex
Size	118 units	105 units	60 units	450 units	600 units	216 units
Ownership	Private – market priced rental	Private – market priced rental	City owned subsidized rental housing	Resident-owned trailers on rented land	Private – market priced	Private – market priced
**Partner Description**						
Partner	Midsize Property owner -	Large Property owner	Municipally owned housing	Residents Board and Recreation Board	Large Property owner	Large Property owner
Primary Contact	Regional Manager	Assistant Vice-president	Manager of Support Services	Residents Board Chair	Assistant Vice-president	Regional Manager
**Resident characteristics and engagement**						
Age density*	40%	40%	35%	50%	30%	25%
Marginalization	Low	Medium	High	Medium	High	Medium
Initial Resident Contact(s)	Members of Social Committee (self-referred)	Small group of residents (landlord referred)	Small group of residents (landlord referred)	Large group of residents (20 +)	Information table in lobby	Small group of residents (landlord referred)
**Environment**						
Internal amenities	Small common room with kitchenette	Large common room, kitchen, dedicated lounge, pool	Small common room with kitchen, patio, garden	Large recreation hall with full kitchen	2 large common rooms	2 large common rooms with kitchenettes
Walk Score** /100)	61	63	57	2	23	51
External amenities (distance from site in km)	Grocery (0.7), Bus stop (0.18), Mall (0.65), Seniors Centre (7.6), Library (0.7)	Grocery (1.1 km), Bus stop (0.21), Mall (6.7), Seniors Centre (2.9), Library (3.2)	Grocery (1.0), Bus stop (0.15), Mall (6.2), Seniors Centre (2.2), Library (0.3)	Grocery (7.2), No public transit, Mall (15.5), Seniors centre (16.5), Library (8.0)	Grocery (0.85), Bus stop (0.07), Mall (2.1), Seniors Centre (6.9), Library (4.6)	Grocery (1.5 km), Mall (1.0 km), Bus stop (0.2), Seniors Centre (3.6), Library (1.6)

*Note:* *Age density = % of residents ≥ 55 years. **Walk Score – higher score = greater walkability of neighbourhood [50].

## Data Availability

Data used in this study included publically available data (Census Canada Data through Statistics Canada) and study specific data related to Oasis participant and site descriptions. Data used to identify and describe NORCs for Oasis programming can be accessed at https://www150.statcan.gc.ca/n1/en/catalogue/92-169-X and https://www12.statcan.gc.ca/census-recensement/2016/dp-pd/prof/index.cfm?Lang=E Anonymized data related to participant and site characteristics are available from the corresponding author on reasonable request.

## Abbreviations

DA: Dissemination area
NORC: Naturally occurring retirement community
NORC-SSPs: Naturally occurring retirement communities with targeted support service programming
SSP: Supportive service programming
